# Temporal Dynamics of Endothelium After Radiation Injury Reveal a Transient Pro-Angiogenic Capillary Subpopulation Associated with Skin Repair

**DOI:** 10.3390/ijms27062879

**Published:** 2026-03-22

**Authors:** Xuejiao Ren, Yating Cai, Chengming Gao, Yifei Qiu, Xia Wang, Huiyang Song, Yansheng Zhu, Xiaoqi Zhou, Jianhao Li, Gangqiao Zhou, Pengbo Cao

**Affiliations:** 1 Collaborative Innovation Center for Personalized Cancer Medicine, Center for Global Health, School of Public Health, Nanjing Medical University, Nanjing 211166, China; renxjy@163.com (X.R.); c_yting@126.com (Y.C.); 2 State Key Laboratory of Medical Proteomics, National Center for Protein Sciences at Beijing, Beijing 102206, China; gchengming1988@163.com (C.G.); 3180101060@zju.edu.cn (Y.Q.); 17776836272@163.com (X.W.); a1291166857@163.com (H.S.); 3 Academy of Military Medical Sciences, Beijing 100850, China; 4 College of Life Sciences, Anhui Medical University, Hefei 230032, China; yszhu810@163.com; 5 College of Life Science, Hebei University, Baoding 071002, China; 15164542322@139.com; 6 Hengyang Medical College, University of South China, Hengyang 421001, China; 13986762047@163.com

**Keywords:** ionizing radiation, skin, scRNA-seq, vascular endothelial cells, Sp1

## Abstract

Ionizing radiation (IR) causes severe vascular damage, yet the dynamic functional states and regulatory mechanisms of vascular endothelial cells (VECs) after irradiation remain poorly understood. To elucidate the underlying processes, we analyzed single-cell RNA sequencing data from mouse dorsal skin collected at multiple post-irradiation (p.i.) time points using trajectory inference, pathway enrichment, transcription factor activity inference, and cell–cell communication analyses. Our results showed that VECs exhibited marked temporal dynamics after irradiation, transitioning from early-stage stress responses to middle-stage angiogenic remodeling and late-stage restoration of homeostasis. A transient *Gpihbp1*^+^ capillary endothelial subpopulation (capVEC2) emerged predominantly during the middle stage (2–3 days p.i.) and was enriched for angiogenesis- and migration-related programs. Enhanced Sp1 regulatory activity was associated with its pro-angiogenic phenotype. At 2 days p.i., capVEC2 engaged in pro-angiogenic and pro-repair signaling with keratinocytes, whereas by 3 days p.i. these interactions shifted toward immune surveillance and tissue homeostasis, accompanied by increased pro-inflammatory and pro-apoptotic signaling and a decline in capVEC2 abundance. Collectively, our findings identify a radiation-induced, transient functional endothelial subpopulation that is associated with vascular–epidermal communication during skin repair post irradiation.

## 1. Introduction

Ionizing radiation (IR) is a high-energy form of radiation that induces characteristic tissue injury, resulting in structural and functional alterations in normal tissues. At the cellular level, ionizing radiation causes DNA damage either directly or indirectly through the generation of reactive oxygen species (ROS), leading to DNA strand breaks and the activation of apoptotic pathways [1]. Tissues with high proliferative activity, including the skin, bone marrow, and gastrointestinal tract, are particularly susceptible to ionizing radiation-induced injury [2].

With the expanding clinical application of ionizing radiation in nuclear medicine and radiotherapy, radiation exposure represents a growing concern for human health. Notably, about 95% of cancer patients receiving radiotherapy develop radiation dermatitis [3,4]. As the largest organ of the human body, the skin is frequently among the first tissues exposed to and affected by ionizing radiation. The extensive and highly organized vascular network within the skin places vascular endothelial cells as key mediators of radiation-induced tissue injury.

Endothelial cells are highly specialized and dynamic regulators of tissue homeostasis rather than merely passive structural components of the blood vessels. By integrating metabolic regulation, cytokine signaling, and immune cell trafficking, endothelial cells coordinate vascular function across a broad range of physiological and pathological contexts. In the skin, endothelial cell-mediated angiogenesis is essential for wound healing and hair follicle cycling [5,6]. Moreover, skin endothelial cells serve as a critical interface between the circulation and the tissue microenvironment, modulating local inflammatory responses through the regulation of immune cell recruitment, activation, and effector functions.

Endothelial dysfunction represents a central pathological event in radiation-induced tissue injury [7]. Microvascular endothelial cells exhibit pronounced radiosensitivity and undergo structural and functional alterations following irradiation. In radiation-induced skin injury, radiation-induced endothelial dysfunction is associated with impaired granulation tissue formation and delayed wound healing [8], highlighting the role of endothelial cells in radiation responses. Recent advances in single-cell RNA sequencing (scRNA-seq) have enabled systematic profiling of cellular composition and molecular responses in radiation-injured skin. Cross-species scRNA-seq of irradiated human and rat skin have revealed shifts in fibroblast and keratinocyte populations and identified the nuclear receptor *Nur77* as a key regulator of radiation-induced skin injury [9]. Additional studies have reported radiation-induced activation of cycling keratinocytes, secretory-papillary fibroblasts, and lymphatic endothelial cells that interact with immune cells to influence injury progression [10]. However, most studies of radiation-injured skin have focused on global microenvironmental alterations or dominant epithelial and stromal populations, whereas skin vascular endothelial cells have largely been examined at the bulk level, leaving their temporal dynamics and functional regulation poorly defined [11,12,13].

Therefore, we applied scRNA-seq to mouse skin collected at multiple time points after irradiation to characterize endothelial heterogeneity and temporal dynamics. Integration of trajectory inference, transcription factor activity inference, and cell–cell communication analyses revealed dynamic transcriptional changes in endothelial cells that may underlie skin repair following irradiation.

## 2. Results

### 2.1. Multi-Temporal scRNA-Seq Reveals Dynamic Changes in Skin Endothelial Cells Following Irradiation

**Figure 1:**
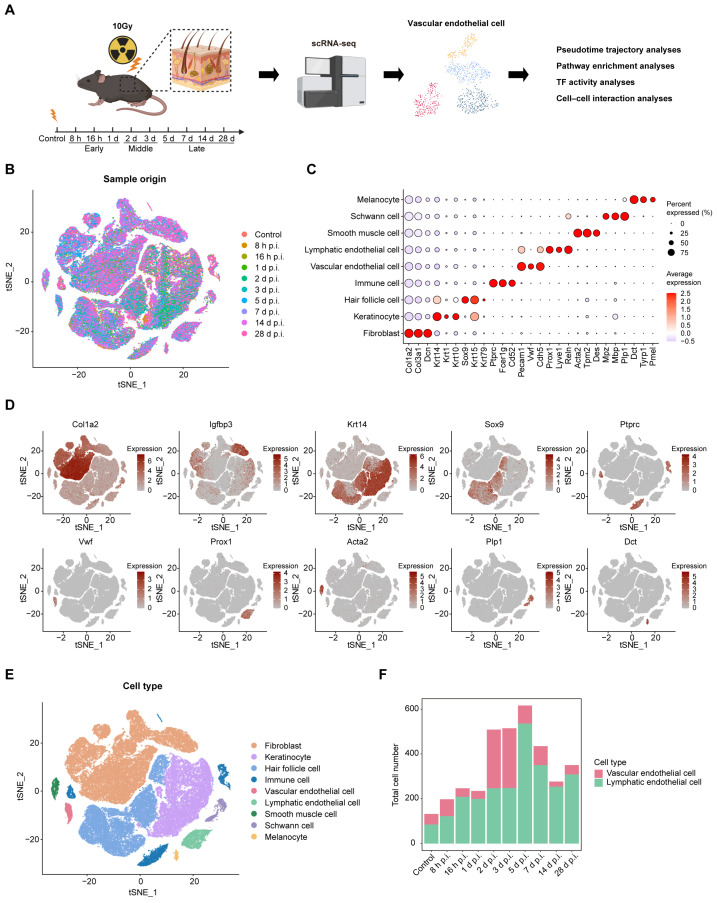
Overview of the single-cell landscape for mouse dorsal skin post irradiation. (**A**) Schematic diagram of the experimental design and scRNA-seq analysis workflow. (Created with bioRender.com). (**B**) t-distributed Stochastic Neighbor Embedding (t-SNE) plot colored by sample origin. (**C**) Dot plot showing percentage of expressed cells and average expression levels of canonical marker genes of the 9 major cell types. (**D**) Feature plots of selected typical canonical markers of each major cell type. (**E**) t-SNE plot showing the transcriptomic landscape of 85,358 high-quality cells, which were classified into nine major cell types. Cells are colored by cell type. (**F**) Stacked bar plot showing the total numbers of vascular endothelial cells and lymphatic endothelial cells at different time points post irradiation.

To characterize skin cellular responses to radiation injury, we performed scRNA-seq on dorsal skin tissues collected from mice exposed to localized ionizing radiation. Mice received a single dose of 10 gray (Gy) dorsal irradiation using a ^60^Co irradiator, with sham-irradiated mice serving as controls. Dorsal skin samples were collected from control mice and irradiated mice at 8 h, 16 h, and 1, 2, 3, 5, 7, 14, and 28 days post-irradiation (p.i.) for downstream single-cell transcriptomic analysis (Figure 1A). After data integration and quality control, 85,358 high-quality cells were retained for downstream analyses. Batch effects across samples were effectively corrected using Harmony, resulting in robust integration of cells from all time points (Figure 1B). Dimensionality reduction and visualization using t-distributed Stochastic Neighbor Embedding (t-SNE) identified nine major cell types based on canonical marker gene expression [14,15] (Figure 1C–E). These included fibroblasts (e.g., *Col1a2* and *Col3a1*), with a dermal papilla subpopulation marked by high *Igfbp3* expression; keratinocytes (e.g., *Krt14* and *Krt10*); hair follicle cells (e.g., *Sox9* and *Krt15*); immune cells (e.g., *Ptprc* and *Fcer1g*); vascular endothelial cells (e.g., *Pecam1* and *Vwf*); lymphatic endothelial cells (e.g., *Pecam1* and *Prox1*); smooth muscle cells (e.g., *Acta2* and *Tpm2*); Schwann cells (e.g., *Plp1* and *Mpz*); and melanocytes (e.g., *Dct* and *Tyrp1*).

We next examined the temporal dynamics of endothelial cell abundance following irradiation (Figure 1F). Vascular endothelial cells exhibited a pronounced increase at 2 and 3 days p.i., followed by a gradual decline, returning to near-baseline levels by 28 d p.i. In contrast, lymphatic endothelial cells showed a sustained increase across all time points, with relative peaks observed at 5 d and 7 d p.i.

Collectively, these results define the major cellular composition of mouse skin and reveal pronounced, time-dependent changes in endothelial cell populations following irradiation, suggesting a potential role for endothelial cells in coordinating radiation-induced skin injury and repair.

### 2.2. Vascular Endothelial Cells Undergo Pronounced Three-Stage Compositional Remodeling Following Irradiation

The skin endothelium exhibits pronounced anatomical and functional heterogeneity. Skin lymphatic vessels are organized into superficial and deep lymphatic plexuses, in which superficial lymphatics are primarily capillary vessels mediating tissue fluid drainage and immune cell trafficking, whereas deep lymphatic plexuses contain collecting vessels that drain into regional lymph nodes [16]. In parallel, the skin blood vasculature comprises distinct arterial, capillary, and venous compartments that support nutrient exchange, inflammatory responses, and tissue repair [17]. Given the fundamental structural and functional differences between lymphatic and vascular endothelium, we first characterized endothelial cell heterogeneity in irradiated skin.

**Figure 2:**
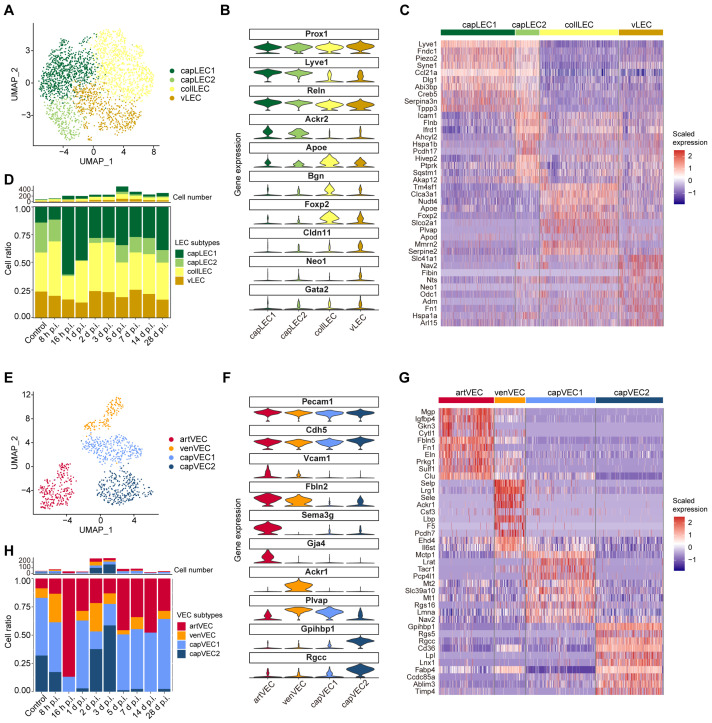
Endothelial cell heterogeneity and temporal dynamics post irradiation. (**A**) Uniform Manifold Approximation and Projection (UMAP) plot of scRNA-seq profile from lymphatic endothelial cells (LECs) which are separated into 4 cell clusters. Cells are colored according to different clusters. (**B**) Violin plot showing the expression of representative marker genes across LEC subpopulations. (**C**) Heatmap showing the scaled expression of the top 10 marker genes for each LEC subpopulation. (**D**) Changes in the absolute numbers and relative proportions of LEC subpopulations across different time points post irradiation. The upper panel shows the total cell numbers, and the lower panel shows the relative proportions of capLEC1, capLEC2, collLEC, and vLEC. (**E**) UMAP plot of scRNA-seq profile from vascular endothelial cells (VECs) which are separated into 4 cell clusters. Cells are colored according to different clusters. (**F**) Violin plot showing the expression of representative marker genes across VEC subpopulations. (**G**) Heatmap showing the scaled expression of the top 10 marker genes for each VEC subpopulation. (**H**) Changes in the absolute numbers and relative proportions of VEC subpopulations across different time points post irradiation. The upper panel shows the total cell numbers, and the lower panel shows the relative proportions of artVEC, venVEC, capVEC1, and capVEC2.

Lymphatic endothelial cells (LECs) were resolved into four subpopulations, including capillary lymphatic endothelial cells (capLEC1 and capLEC2), collecting lymphatic endothelial cells (collLEC), and valve lymphatic endothelial cells (vLEC) (Figure 2A–C and Figure S1A). All LEC subtypes robustly expressed the pan-lymphatic marker *Prox1* [18]. capLEC1 and capLEC2 were characterized by high expression of *Lyve1*, *Reln*, and *Ackr2*; collLECs expressed *Apoe*, *Bgn*, and *Foxp2*; vLECs exhibited high expression of *Cldn11* and *Neo1*, consistent with previously reported transcriptional features of lymphatic endothelial subtypes [19,20]. Further analyses revealed no significant alterations in the relative proportions of lymphatic endothelial subpopulations after irradiation across all time points (Figure 2D and Figure S1B), indicating relative stability of the lymphatic endothelium following irradiation.

Vascular endothelial cells (VECs) were classified into four subpopulations, including arterial endothelial cells (artVEC), venous endothelial cells (venVEC), and two capillary endothelial subsets (capVEC1 and capVEC2) (Figure 2E–G and Figure S1C). All VEC subtypes expressed the canonical pan-endothelial markers *Pecam1* and *Cdh5*. artVEC were defined by expression of the arterial marker *Sema3g* and the gap junction gene *Gja4*, whereas venVEC specifically expressed *Ackr1*, which has been reported to be highly expressed in post-capillary venules and venules but not arterioles [21]. Both artVEC and venVEC expressed *Fbln2*, a gene associated with collagen-containing extracellular matrix and elastic intima [5]. capVEC1 exhibited high *Plvap* expression, indicative of fenestrated capillary endothelium, whereas capVEC2 expressed the capillary markers *Gpihbp1* and *Rgcc*, consistent with capillary endothelial identity [22].

To assess the impact of irradiation on vascular endothelial composition, we examined temporal changes in VEC subpopulations. Among these, capVEC2 exhibited the most pronounced dynamic changes (Figure 2H and Figure S1D). Specifically, the abundance of capVEC2 decreased during the early stage (8 h-1 d p.i.), markedly increased during the middle stage (2–3 d p.i), and declined again during the late stage (5–28 d p.i.), forming a distinct three-stage pattern of decrease-increase-decrease. venVEC and capVEC1 exhibited similar three-stage quantitative trends following irradiation (Figure 2H). In contrast, artVEC proportions remained relatively stable, and their apparent changes primarily reflected relative shifts driven by alterations in venous and capillary endothelial populations (Figure S1D). This observation is consistent with the limited proliferative capacity and characteristic cell-cycle arrest of arterial endothelial cells [23]. To further support the stage classification, we applied Milo differential abundance analysis [24]. In the comparison between the early and middle stages, neighborhoods corresponding to capVEC2 showed significant increases in abundance, whereas other endothelial subpopulations exhibited relatively minor changes (Figure S2A,B). In contrast, comparison between the middle and late stages revealed a marked decrease in capVEC2-associated neighborhoods, consistent with the decline in capVEC2 abundance observed in the late stage (Figure S2C,D). These results further support the stage-dependent dynamics of endothelial subpopulations following irradiation.

In summary, these results reveal the heterogeneity of lymphatic and vascular endothelium, and indicate that vascular endothelial cells undergo coordinated early-, middle-, and late-stage changes following radiation injury. These observations provide a basis for downstream functional analyses.

### 2.3. Three-Stage Trajectory Analyses Reveal a capVEC2-Specific State Transition During the Middle Stage

**Figure 3:**
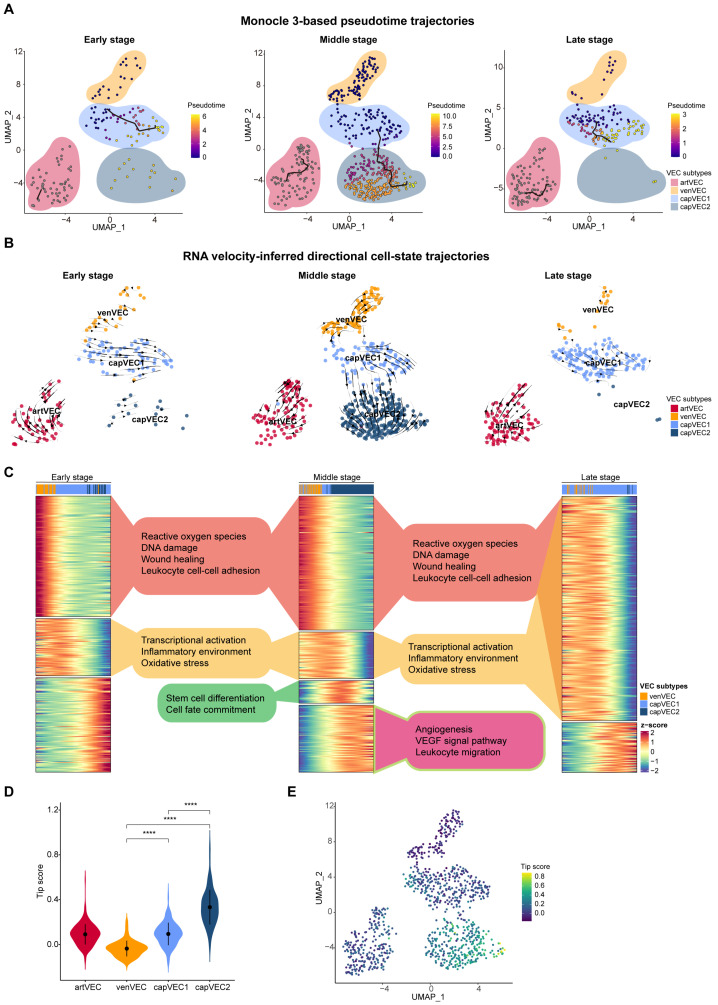
Dynamic vascular endothelial cell state transitions post irradiation. (**A**) Monocle 3-based pseudotime trajectories of vascular endothelial cells at early, middle, and late stages post irradiation, visualized on UMAP plots. The black curve represents the inferred trajectory constructed by Monocle3, indicating the progression of cells along pseudotime. Cells are colored by pseudotime values, and inferred developmental trajectories are indicated. early stage (8 h–1 d p.i.), middle stage (2 d–3 d p.i.), and late stage (3 d–28 d p.i.). (**B**) RNA velocity-inferred directional cell-state trajectories of vascular endothelial cells at early, middle, and late stages post irradiation. Velocity vectors indicate the predicted future transcriptional states of individual cells. (**C**) Heatmaps showing dynamic changes in gene expression programs along pseudotime at early, middle, and late stages post irradiation. Representative Gene Ontology (GO) terms associated with each expression pattern are shown in the middle. (**D**) Violin plots showing the distribution of tip scores across VEC subpopulations. The tip score is calculated based on a published tip cell gene signature and reflects tip cell–like transcriptional characteristics. Statistical significance was assessed using the Wilcoxon rank-sum test. **** *p* < 0.0001. (**E**) UMAP plot colored by tip score.

To investigate radiation-induced changes in vascular endothelial cell states, we performed trajectory analysis of vascular endothelial cells across three stages using Monocle 3 [25] (Figure S3A). Based on transcriptional similarity, vascular endothelial cells segregated into two largely independent pseudotime trajectories, one corresponding to arterial endothelial cells, and the other comprising venous and capillary endothelial populations (Figure 3A). Comparison of trajectory structures across the three stages revealed distinct stage-specific dynamics. During the early stage, a transition from venVEC to capVEC1 was observed, consistent with an acute endothelial response to radiation injury (Figure 3A). During the middle stage, the trajectory architecture was markedly reorganized, with the emergence of a continuous pseudotime transition from venVEC through capVEC1 to capVEC2, reflecting increased state plasticity and active capillary endothelial remodeling (Figure 3A). In contrast, during the late stage, pseudotime dynamics were largely confined the capVEC1 subpopulation, indicating a return toward relative stability in endothelial cell states (Figure 3A).

To validate these trajectory inferences, we independently examined endothelial transcriptional dynamics using RNA velocity analyses [26] (Figure 3B). Consistently, RNA velocity vectors during the middle stage exhibited a clear directional flow from venVEC through capVEC1 toward capVEC2 (Figure 3B). During the early stage, velocity vectors predominantly pointed from venVEC toward capVEC1, whereas during the late stage, they were largely confined within the capVEC1 compartment (Figure 3B). Slingshot trajectory analysis [27] further supported these observations (Figure S3B). Collectively, results from multiple complementary approaches demonstrate that vascular endothelial cell state transitions following irradiation occur in a stage-dependent manner, with the most pronounced remodeling observed during the middle stage.

### 2.4. capVEC2 Represents a Transient, Tip-like Angiogenic Endothelial Cells After Radiation

Building on the pseudotime trajectories of vascular endothelial cells, we next examined the pseudotime-associated functional programs inferred by Monocle 3 (Figure 3A). Finaly, we identified distinct transcriptional modules with stage-specific expression patterns that define functional programs of endothelial cell subpopulations following irradiation (Figure 3C).

During the early stage, pseudotime-associated genes were resolved into three distinct functional modules (Figure S3C). Module 1, primarily composed of venVEC, was characterized by the activation of DNA damage response, oxidative stress, wound healing, and leukocyte recruitment, consistent with an acute stress and inflammatory reaction (Figure S3C). In contrast, module 2, dominated by capVEC1, preferentially engaged transcriptional regulatory programs alongside inflammatory and oxidative stress responses, suggesting a coordinated transcriptional adaptation to injury (Figure S3C). Module 3 was positioned at a later segment of the pseudotime trajectory and was composed of both capVEC1 and capVEC2 cells. This module was enriched for cell migration, indicative of early endothelial mobilization (Figure S3C). Collectively, these features align well with previous observations that irradiation rapidly induces a pro-inflammatory endothelial phenotype accompanied by enhanced immune cell recruitment [7,28,29].

As the response progressed into the middle stage, pseudotime-associated genes segregated into four functional modules, revealing increased complexity in endothelial state transitions (Figure S3D,E). Modules 1 and 2 largely overlapped with their early-stage counterparts (Figure S3D). Module 3 captured a key transitional window along the trajectory from capVEC1 toward capVEC2 and was specifically enriched for pathways involved in cell fate determination and stem cell differentiation (Figure S3D), reflecting ongoing phenotypic reprogramming. Notably, module 4, positioned at the distal end of the pseudotime axis and dominated by capVEC2, exhibited strong enrichment for angiogenesis and cell migration pathways (Figure S3D,E), emphasizing this stage as a phase of active vascular remodeling and regenerative expansion.

By the late stage, pseudotime-associated genes converged into two major modules, both predominantly composed of capVEC1 cells (Figure S3F). These modules were enriched for pathways related to transcriptional regulation, wound healing, oxidative stress response, and DNA damage repair (Figure S3F). These features indicate a transition from an inflammatory and stress-associated state toward restoration of genomic stability and endothelial maintenance.

Integration of functional modules across all stages revealed clear subpopulation-specific specialization during the radiation response (Figure 3C). venVEC was predominantly enriched for DNA damage response and oxidative stress pathways during the early stage, consistent with an acute injury-sensing role (Figure 3C). capVEC1 displayed sustained enrichment for antioxidant defenses and transcriptional regulatory programs across stages, consistent with a role in supporting endothelial survival and homeostatic maintenance under prolonged stress (Figure 3C). In contrast, capVEC2 emerged specifically during the later part of the middle stage, showed strong enrichment for angiogenesis and migration-related pathways, and was largely absent at the late stage (Figure 3C). These features indicate that capVEC2 represents a transient, radiation-induced endothelial state associated with vascular remodeling and regeneration.

During angiogenesis, tip cells sense and respond to guidance cues and lead vascular sprouting through directed migration, whereas stalk cells follow to support sprout elongation and lumen formation [30]. Based on the transcriptional features of capVEC2, we indicate that this subpopulation represents a radiation-induced endothelial state with tip cell-like characteristics in the skin. A recent large-scale single-cell analysis of tumor vasculature published in Nature identified *APLN*^+^ endothelial cells as a canonical tip cell population during angiogenesis [31]. Using the tip cell signature genes defined in this study, we Found that capVEC2 displayed significantly higher tip signature scores than the other endothelial subpopulations (Figure 3D,E). Collectively, these results support capVEC2 as a radiation-induced, tip-like endothelial state that may contribute to angiogenic responses and vascular remodeling in irradiated skin.

### 2.5. Sp1 Is Identified as a Key Transcription Factor Associated with the Angiogenic State of capVEC2

**Figure 4:**
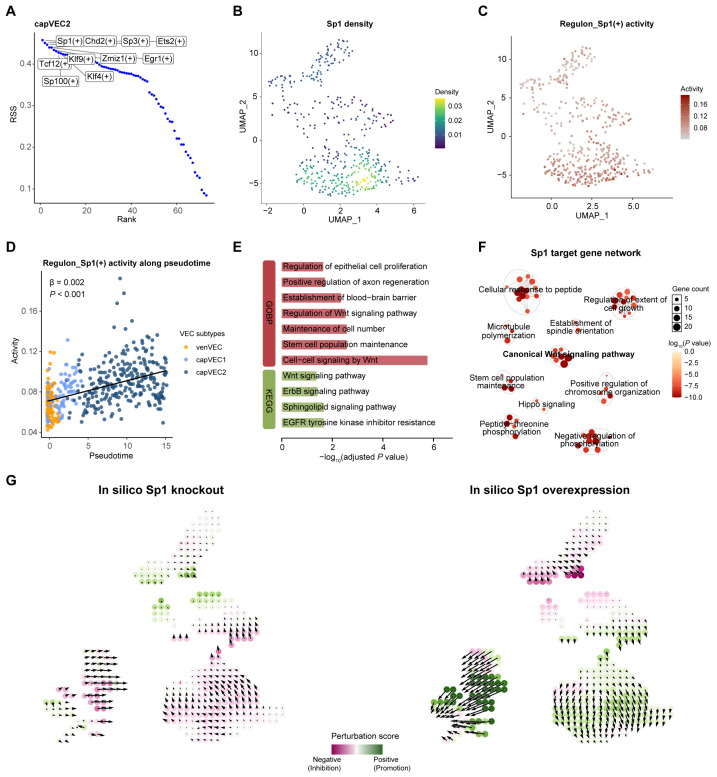
*Sp1*-dependent transcriptional regulation in capVEC2 during the middle stage. (**A**) Scatter plots of top 10 transcription factor (TF) regulons in capVEC2 ranked by regulon specificity score (RSS). (**B**) UMAP plot showing the *Sp1* expression levels in vascular endothelial cells. (**C**) UMAP plot showing the *Sp1* regulon activity in vascular endothelial cells. (**D**) *Sp1* regulon activity along pseudotime. Cells are colored by VEC subpopulation. (**E**) Pathway enrichment of *Sp1* regulon target genes. (**F**) Network visualization of enriched pathways associated with *Sp1* target genes. Each node represents an enriched pathway, with node size indicating the number of genes and node color reflecting enrichment significance. Edges indicate shared genes between pathways. (**G**) Perturbation simulation vector fields under in silico *Sp1* knockout (**left**) or overexpression (**right**). Positive (green) and negative (purple) perturbation scores indicate promotion and inhibition of cell state changes, respectively. Arrows represent the predicted direction and magnitude of cell state transitions inferred by the model.

To elucidate the molecular mechanisms underlying the angiogenic state of capVEC2, we systematically characterized transcription factor (TF) regulatory networks in vascular endothelial cells using SCENIC analyses [32]. capVEC2 showed significant enrichment of TFs, including *Klf4*, *Sp1*, *Sp3*, and *Ets2* (Figure 4A and Figure S4A). *Klf4* has been implicated in endothelial homeostasis [33,34], whereas *Sp1*, *Sp3*, and *Ets2* are well-established transcriptional regulators of angiogenesis. Consistent with this role, endothelial-specific *Sp1*/*Sp3* knockout reduces angiogenesis in retinal, pathological, and tumor models [35,36]. *Ets2* promotes capillary growth through transcriptional activation of VEGFA [37,38,39]. Correlation analysis revealed strong co-activation among *Sp1*, *Sp3*, and *Ets2* regulons (Figure S4B), indicating a coordinated pro-angiogenic transcriptional network in capVEC2.

In contrast, other vascular endothelial subpopulations displayed distinct TF enrichment profiles during the middle stage. artVEC was enriched for *Msx1*, a regulator of arterial development and vascular remodeling [40], together with the oxidative stress-associated TF *Nrf1* [41] (Figure S4C). venVEC showed enrichment of the pro-inflammatory TF *Bcl3* and the oxidative stress-responsive TFs *Atf4* and *Atf6* [42,43,44,45] (Figure S4D), while capVEC1 was characterized by enrichment of *Atf6* and the inflammatory TFs *Irf1* and *Irf7* (Figure S4E). These TF signatures closely paralleled the functional states identified by pseudotime analyses.

Given the pronounced angiogenic phenotype of capVEC2 during the middle stage, we next sought to identify core transcriptional regulatory drivers within this subpopulation. Among candidate transcription factors, *Sp1* displayed the most prominent increase in regulon activity in capVEC2, accompanied by elevated *Sp1* expression levels (Figure 4B,C and Figure S4F). Analysis of *Sp1* regulon dynamics along the pseudotime trajectory revealed sustained and progressive activation of *Sp1* toward the terminal, capVEC2-enriched phase of the trajectory (Figure 4D). Pathway enrichment analysis of *Sp1* target genes revealed significant enrichment for pathways related to angiogenesis (e.g., *Pdgfd*, *Stat3*, *Rptor* and *Sav1*), stem cell population maintenance (e.g., *Stat3*, *Sav1* and *Braf*), and Wnt signaling (e.g., *Wls*, *Lrp6* and *Macf1*) (Figure 4E). Network analyses based on Gene Ontology (GO) enrichment of *Sp1* target genes further revealed interconnected functional modules (Figure 4F). These modules included pathways related to canonical Wnt signaling, regulation of cell growth, stem cell population maintenance, and establishment of spindle orientation, supporting a potential involvement of *Sp1* in endothelial proliferation and vascular remodeling. In summary, *Sp1* may represent a key transcription factor associated with the angiogenic and vascular regenerative state of capVEC2.

To better understand the impact of *Sp1* on the angiogenic states of capVEC2 during the middle stage, we performed in silico perturbation analysis using CellOracle [46]. We found that simulated *Sp1* knockout led to an attenuation of the angiogenic state in capVEC2, whereas *Sp1* overexpression promoted angiogenic features within this subpopulation (Figure 4G). Collectively, these results support a potential role for *Sp1* as a key TF associated with the establishment and maintenance of the angiogenic endothelial state of capVEC2 during the middle stage.

### 2.6. Time-Dependent Angiogenic and Cell Fate Dynamics of capVEC2

The angiogenic endothelial state of capVEC2 is not only dependent on cell-intrinsic transcriptional programs but is also shaped by intercellular signaling within the tissue microenvironment. We therefore further investigated the regulatory mechanisms underlying this phenotype from the perspective of intercellular communication. First, we performed ligand-to-target signaling analyses between the capVEC2 (receiver) and all other cell types (senders) at 2 d and 3 d p.i. using NicheNet [47].

**Figure 5:**
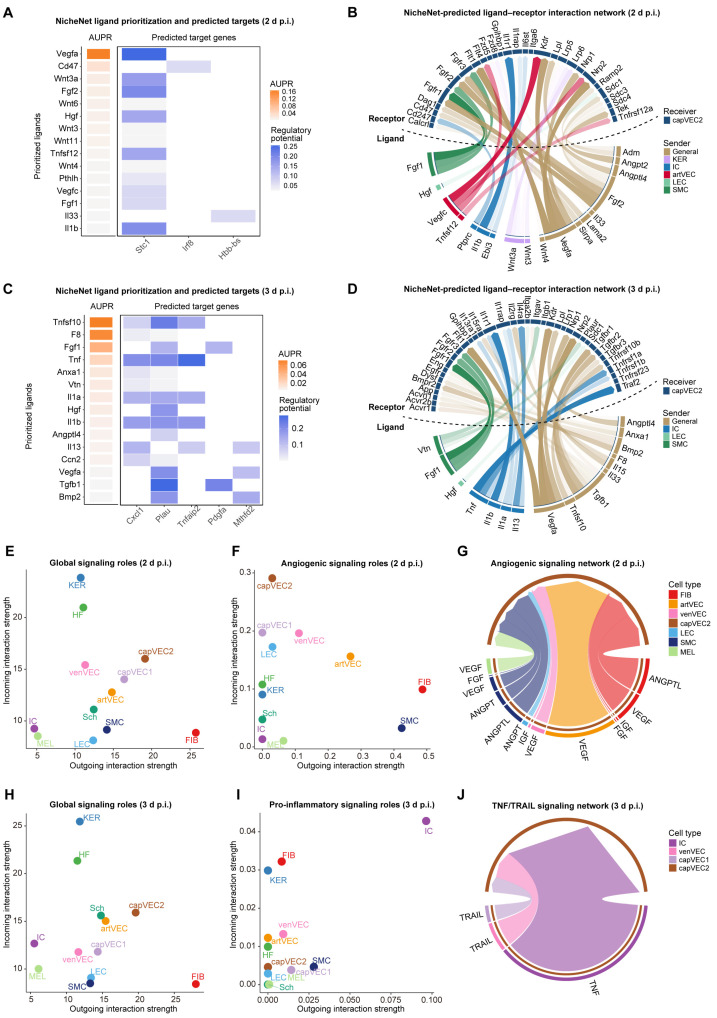
Cell–cell communication patterns with capVEC2 as the receiver during the middle stage. (**A**) Ligand-to-target signaling analysis for capVEC2 (receiver) and surrounding cell clusters (senders) at 2 d p.i. using the NicheNet framework. Prioritized ligands (left) and predicted target genes in capVEC2 (right) are shown. AUPR, area under the precision–recall curve. (**B**) Circos plot showing top ligand–receptor interactions between sender and capVEC2 (receiver) at 2 d p.i. (**C**) Ligand-to-target signaling analysis for capVEC2 (receiver) and surrounding cell clusters (senders) at 3 d p.i. (**D**) Circos plot showing top ligand–receptor interactions between sender and capVEC2 (receiver) at 3 d p.i. (**E**) Scatter plot showing global signaling roles of cell types at 2 d p.i., defined by outgoing (sender) and incoming (receiver) interaction strengths. (**F**) Scatter plot showing angiogenic signaling roles of cell types at 2 d p.i. (**G**) Circos plot showing the angiogenic signaling network at 2 d p.i. (**H**) Scatter plot showing global signaling roles of cell types at 3 d p.i. (**I**) Scatter plot showing pro-inflammatory signaling roles of cell types at 3 d p.i. Slight jittering was applied to overlapping points for visualization purposes. (**J**) Circos plot showing the TNF/TRAIL signaling network at 3 d p.i. Throughout this figure, FIB denotes fibroblast; KER, keratinocyte; HF, hair follicle cell; IC, immune cell; artVEC, arterial vascular endothelial cell; venVEC, venous vascular endothelial cell; capVEC1/2, capillary vascular endothelial subtypes 1 and 2; LEC, lymphatic endothelial cell; SMC, smooth muscle cell; Sch, Schwann cell; MEL, melanocyte.

At 2 d p.i., ligand activity prediction predicted increased activity of canonical pro-angiogenic factors, including *Vegfa*, *Fgf2*, *Vegfc*, and *Fgf1*, together with the Wnt ligand *Wnt3a* (Figure 5A). Among the predicted target genes, *Stc1* emerged as a highly regulated target and has been previously implicated in angiogenic processes [48]. Consistent with this pro-angiogenic profile, ligand–receptor interaction analyses suggested the presence of multiple angiogenesis-related signaling pathways (Figure 5B). *Vegfa*, which exhibited the highest regulatory potential score at 2 d p.i., is a central regulator of angiogenesis [49]. The predicted ligand-receptor interaction between Vegfa and its receptor Kdr expressed in capVEC2 suggests that VEGF signaling may be associated with the angiogenesis-related features observed in this subpopulation. (Figure 5B). In addition, ANGPT, FGF [50], and Wnt [51] signaling pathways were also predicted to be active, further suggesting angiogenesis-associated features in capVEC2 (Figure 5B). Together, these features are consistent with the transient expansion of capVEC2 observed at 2 d p.i. (Figure 2H), suggesting that capVEC2 may predominantly exhibit angiogenesis-related features at this stage.

By contrast, at 3 d p.i., the predicted ligand profile shifted toward inflammatory and cell fate–associated signals. The top-ranked ligands included the apoptosis-related ligand *Tnfsf10* as well as pro-inflammatory cytokines such as *Tnf*, *Il1a*, *Il1b*, and *Il13* (Figure 5C), suggesting that capVEC2 may be exposed to an inflammatory signaling environment with pro-apoptotic cues. At the same time, pro-angiogenic ligands including *Fgf1* and *Vegfa* remained predicted (Figure 5C), suggesting the coexistence of angiogenic and inflammatory signals at this stage. Predicted target genes at 3 d p.i. included the inflammatory chemokine *Cxcl1*, the TNF-responsive gene *Tnfaip2*, and *Plau* (Figure 5C), which has been reported to regulate inflammatory responses and endothelial cell migration [52]. Ligand–receptor interaction analyses (Figure 5D) further indicated the persistence of angiogenic signaling alongside the emergence of strong pro-apoptotic and pro-inflammatory pathways. Among these, *Tnfsf10*, which exhibited the highest regulatory potential score at 3 d p.i., is a well-established inducer of apoptosis. Binding of *Tnfsf10* to its receptor *Tnfrsf10b* (death receptor 5, DR5) on capVEC2 activates caspase-dependent apoptotic pathways [53,54]. In addition to this core pro-apoptotic signal, pro-inflammatory cytokines such as TNF and IL-1 have been reported to enhance TRAIL-mediated apoptotic signaling [55]. Moreover, the predicted ligand-receptor interaction between Tnf and Tnfrsf1a suggests that capVEC2 may be exposed to apoptosis-related signaling cues [56]. Collectively, these signaling pathways are likely to increase the susceptibility of capVEC2 to apoptotic cues, consistent with the marked decline in capVEC2 abundance observed after 3 d p.i. (Figure 2H). These findings support a model in which capVEC2 shifts from a predominantly angiogenic state to an inflammation- and apoptosis-associated state at this time point.

To further explore the NicheNet predictions, we applied CellChat [57] to systematically analyze intercellular communication networks at 2 d and 3 d p.i. (Figure S5A–D). At 2 d p.i., global intercellular communication analysis across all signaling pathways identified fibroblasts as the dominant signal sender, while keratinocytes served as the primary signal receiver (Figure 5E). This pattern is consistent with the physiological organization of skin tissue, in which keratinocytes represent the major cell population and extensively receive signals from mesenchymal cells [58]. Based on the angiogenic features of capVEC2 proposed by NicheNet analyses, we next focused on angiogenic signaling pathways, including VEGF, FGF, ANGPT, ANGPTL, and IGF. The result revealed that capVEC2 was the primary receiver of these signals (Figure 5F). Consistently, chord diagrams illustrated predicted transmission of pro-angiogenic signals from multiple cell types to capVEC2 (Figure 5G). Collectively, these results suggest an intercellular communication pattern consistent with angiogenesis-associated features of capVEC2 at 2 d p.i.

At 3 d p.i., fibroblasts remained prominent signal senders within the microenvironment (Figure 5H). Based on the inflammatory and apoptosis-associated features suggested by the NicheNet analyses, we focused on pro-inflammatory signaling pathways, including TNF, IL-6, and IFN-I. The result showed that capVEC2 predominantly acted as a receiver within these pathways (Figure 5I). In addition, chord diagrams illustrated potential signaling inputs from both TNF and TRAIL to capVEC2 (Figure 5J). To further characterize the temporal transition of capVEC2 signal states, we directly compared intercellular communication patterns between 2 d and 3 d p.i. This analysis revealed the specific emergence of apoptosis-related TRAIL signaling and pro-inflammatory SPP1 signaling at 3 d p.i., accompanied by marked enhancement of TNF and IL2 pathways (Figure S5E). Collectively, these alterations in predicted intercellular communication networks are consistent with a transition of capVEC2 from an angiogenesis-associated state at 2 d p.i. toward an inflammation- and apoptosis-associated transcriptional state at 3 d p.i.

Integrated analyses using NicheNet and CellChat suggest that, during the middle stage, capVEC2 may act as a key receiver of pro-angiogenesis-related signals within the skin microenvironment. At 2 d p.i., capVEC2 appears to receive multiple pro-angiogenic cues, including VEGF, FGF, ANGPT, and Wnt, consistent with the rapid expansion of its angiogenic endothelial state and cell abundance (Figure 2H). At 3 d p.i., apoptosis- and inflammation-related signaling cues become more prominent, suggesting a shift from an angiogenesis-dominant state toward inflammation activation and cell fate-related regulation. This transition is accompanied by a pronounced decline in the capVEC2 subpopulation (Figure S3A).

Together, these observations suggest that capVEC2 may represent a transient functional endothelial state that emerges during the middle stage following irradiation. The inducible expansion of this subpopulation may be associated with angiogenic responses and microenvironmental remodeling, followed by a gradual resolution as these processes subside. These findings raise the question of whether capVEC2 exerts regulatory effects on surrounding cell populations during its transient existence, thereby contributing to the remodeling of the post-irradiation skin microenvironment.

### 2.7. capVEC2–Keratinocyte Communication During Post Irradiation Skin Remodeling

**Figure 6:**
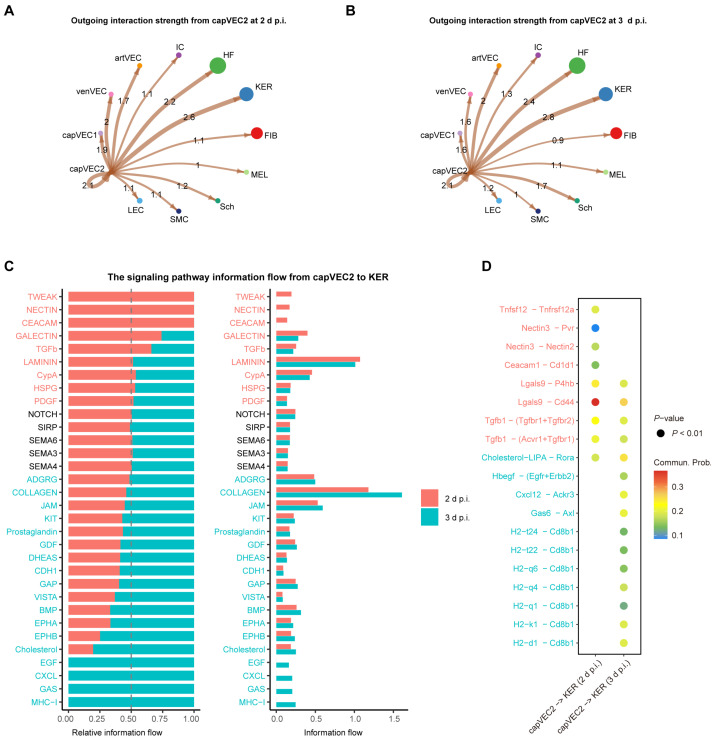
Cell–cell communication patterns with capVEC2 as the sender during the middle stage. (**A**) Outgoing interaction strength from capVEC2 to different cell types at 2 d p.i. Node size indicates cell number, and edge width reflects interaction strength. (**B**) Outgoing interaction strength from capVEC2 to different cell types at 3 d p.i. (**C**) Signaling pathway information flow from capVEC2 to keratinocytes (KER) at 2 d p.i. and 3 d p.i. Left panel shows relative information flow, and right panel shows absolute information flow for each signaling pathway. (**D**) Bubble plot showing ligand–receptor interactions from the top 5 stage-specific signaling pathways at 2 d p.i. (red) and 3 d p.i. (blue), as identified in (**C**). The arrow indicates the direction of signaling from sender (capVEC2) to receiver (KER). Color indicates communication probability, and size indicates statistical significance (*p* < 0.01). Commun. Prob, communication probability. Cell type abbreviations are consistent with those defined in Figure 5.

We next investigated the potential regulatory roles of capVEC2 on other cellular subpopulations. To investigate the interactions between capVEC2 and other cell types, we performed CellChat analyses with capVEC2 as the signal sender. At 2 d p.i., capVEC2 exhibited extensive communication with multiple skin cell subpopulations, with keratinocytes (KER) and hair follicle cells (HF) serving as the primary receivers. Both the number and predicted strength of these interactions were higher, suggesting sustained signaling interactions from capVEC2 to epidermis-associated cell populations during the angiogenic phase (Figure 6A and Figure S6A). At 3 d p.i., communication between capVEC2 and KER as well as HF remained evident, suggesting continued interaction with epidermal cell populations during the inflammatory activation stage (Figure 6B and Figure S6B).

Given the essential role of KER in skin barrier repair and re-epithelialization, together with their consistently strong interactions with capVEC2, we subsequently focused on capVEC2–keratinocyte communication patterns. At 2 d p.i., TWEAK signaling and cell adhesion–related pathways, including NECTIN and CEACAM, were selectively activated, whereas GALECTIN and TGF-β signaling displayed higher activity at 2 d p.i. compared with 3 d p.i. (Figure 6C). These patterns were also replicated in ligand–receptor communication strength analyses (Figure 6D). TWEAK, a member of the TNF superfamily, is known to be induced during skin inflammation or injury and can activate NF-κB signaling to stimulate cytokine production in keratinocytes [59]. Moderate TWEAK activation has been linked to tissue repair, whereas sustained or excessive signaling is associated with tissue damage, fibrosis, and chronic inflammation [60]. In our study, TWEAK signaling was evident at 2 d p.i. and reduced by 3 d p.i. (Figure 6C). This pattern is consistent with a role for TWEAK signaling in supporting early inflammatory and reparative responses, followed by attenuation that helps limit prolonged inflammation and tissue damage. Parallelly, increased NECTIN and CEACAM signaling suggests enhanced vascular–epidermal interactions, which may reflect enhanced signaling interactions between capVEC2 and KER at this stage (Figure 6C). GALECTIN signaling was activated at 2 d p.i. and remained robust at 3 d p.i. (Figure 6C). Consistent with previous studies showing that GALECTIN promotes keratinocyte migration and accelerates re-epithelialization [61,62,63], this sustained activity is consistent with a potential role for GALECTIN signaling throughout the epidermal repair process. Similarly, TGF-β signaling displayed sustained activity from 2 d to 3 d p.i. (Figure 6C), consistent with its role in promoting keratinocyte migration during wound healing [64]. Collectively, the coordinated activation of TWEAK, GALECTIN, TGF-β, and adhesion-related pathways at 2 d p.i. suggests the presence of a vascular–epidermal communication pattern that initiates repair-associated programs while constraining excessive inflammatory and fibrotic responses.

Moreover, MHC-I, GAS, CXCL, and EGF signaling showed more pronounced stage-specific activation at 3 d p.i. than that at 2 d p.i. (Figure 6C). These patterns were also reflected in ligand–receptor interaction analyses (Figure 6D). Notably, MHC-I signaling showed the highest information flow among all pathways at 3 d p.i., suggesting increased involvement in antigen presentation–related signaling by capVEC2 and potentially increased immune surveillance during the repair process. In parallel, several pathways directly linked to epidermal regeneration showed elevated predicted activity. GAS signaling, which has been reported to promote cell survival and migration, inhibit apoptosis, and support stem cell maintenance [65,66], showed strong activity at this stage (Figure 6C). CXCL signaling not only mediates immune cell recruitment but also directly enhances keratinocyte migration and proliferation, thereby accelerating re-epithelialization [67]. EGF signaling, implicated in keratinocyte stem cell expansion and epidermal structural reconstruction [68], was also active at 3 d p.i. (Figure 6C). Cholesterol serves as a key structural component of the epidermis and contributes to the synthesis of steroid hormones, including glucocorticoids, which exert anti-inflammatory and immunosuppressive effects [69]. Accordingly, the specific enhancement of cholesterol signaling may contribute to the modulation of local inflammatory responses and support tissue repair at 3 d p.i. Together, these findings suggest that at 3 d p.i., capVEC2 may interact with KER through coordinated signaling patterns, potentially contributing to a microenvironment associated with epidermal repair and tissue homeostasis.

**Figure 7:**
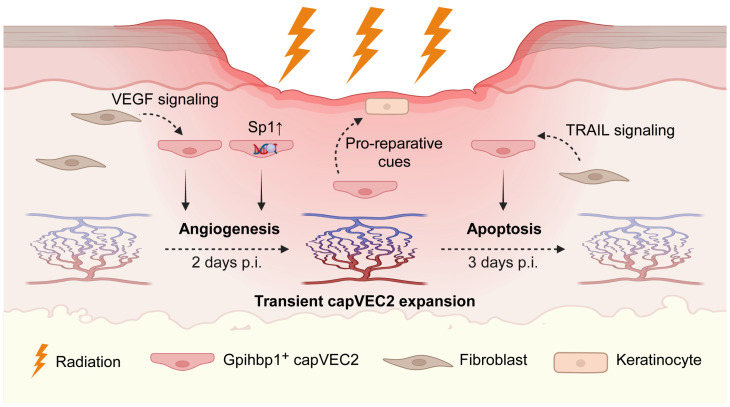
Schematic model illustrating the dynamic role of capVEC2 during radiation-induced skin repair. Dashed arrows denote intercellular signaling or temporal progression, whereas solid arrows indicate cellular responses. (Created in BioRender. REN, J. (2026) https://BioRender.com/8dful4p, accessed on 19 March 2026).

In summary, this study identifies capVEC2 as a potential hub in vascular–epidermal communication that may integrate inflammatory regulation, immune surveillance, and regenerative signaling to shape the epidermal repair microenvironment during skin injury. Based on these findings, we further summarized the temporal dynamics of capVEC2-mediated vascular–epidermal communication following irradiation in a schematic model (Figure 7).

Following irradiation, a transient capillary endothelial subpopulation, *Gpihbp1*^+^ capVEC2, emerges in the dermis and undergoes stage-dependent functional transitions. At 2 d p.i., enhanced VEGF signaling together with increased *Sp1* regulatory activity is associated with angiogenesis-related features and transient expansion of capVEC2, consistent with vascular remodeling and the delivery of pro-reparative cues to the epidermis. By 3 d p.i., capVEC2 exhibits increased sensitivity to apoptotic signaling, particularly TRAIL-associated pathways, accompanied by a decline in the expanded capillary network. Together, this schematic highlights a transient endothelial state in which capVEC2 is associated with angiogenic, apoptotic, and vascular–epidermal signaling patterns during the tissue repair process following radiation injury.

## 3. Discussion

IR exposure is common in clinical settings, particularly during radiotherapy, where radiation-induced tissue injury represents a major clinical complication. Among affected tissues, the skin is especially susceptible, with radiation-induced skin injury frequently manifesting as inflammation, impaired wound healing, and long-term fibrosis [4,70]. During this process, vascular endothelial cells play critical roles in coordinating tissue repair, and radiation-induced endothelial dysfunction can disrupt vascular balance and promote pro-inflammatory responses [1,7,71,72]. Recently, advances in scRNA-seq have substantially expanded our understanding of the cellular and molecular responses underlying radiation-induced skin injury. However, most studies have predominantly focused on epidermal, fibroblast, and immune cell populations, revealing radiation-associated activation of keratinocyte proliferation programs, fibroblast remodeling, and immune-mediated inflammatory responses [9,10]. In contrast, studies specifically focusing on skin vascular endothelial cells following irradiation remain limited. To address this gap, we performed multi-temporal single-cell transcriptomic analyses to systematically dissect the dynamic responses of skin endothelial cells following irradiation. We reveal that vascular endothelial cells undergo distinct stage-dependent responses and identify a unique *Gpihbp1*^+^ capillary endothelial subpopulation, capVEC2. By integrating analyses of pseudotime trajectory, transcriptional regulatory network inference, and intercellular communication, we propose a dynamic functional model in which capVEC2 exhibits inducible emergence, is associated with angiogenic and microenvironment-regulatory functions, and subsequently undergoes programmed regression during post irradiation skin repair.

Together with previous reports, our findings support the idea that radiation exposure may reshapes endothelial transcriptional programs associated with migration and angiogenesis, thereby influencing vascular remodeling. A mouse model of radiation-induced skin injury reported a marked upregulation of vascularization-related genes (e.g., *Hspa1b*, *Neat1*, and *Myl9*) in endothelial cells following irradiation, suggesting that radiation modulates angiogenic responses. Although vascular endothelial subpopulations were not resolved, the vascular endothelial cells as a whole displayed features consistent with enhanced migratory capacity [10]. Building on these observations, our study suggests that vascular endothelial cells undergo a triphasic response to radiation injury and identifies capVEC2 as a transient, radiation-induced endothelial state that expands specifically during the vascular remodeling phase. This population is enriched for angiogenesis- and migration-related programs, suggesting a specialized endothelial state potentially involved in vascular regeneration. Notably, analyses of endothelial heterogeneity in normal human skin have described a capillary endothelial subset characterized by high RGCC expression and enrichment of angiogenesis pathways [5]. Although this population was described under homeostatic conditions, it shares prominent angiogenic features with the Gpihbp1^+^ capVEC2 identified in our injury model, suggesting the presence of a conserved, angiogenic-competent endothelial program across species and physiological states.

At the transcriptional level, integrated SCENIC and in silico perturbation analyses identified *Sp1* as a key transcriptional factor associated with the capVEC2 state. Consistent with this finding, previous studies have found that knockdown of *Sp1* impairs endothelial proliferation and tube formation in vitro [73]; and *Sp1*-depleted mice exhibit markedly reduced retinal vessel area, vascular length, branching points, and sprouting activity [35], highlighting an essential role for *Sp1* in angiogenesis. At the intercellular communication level, combined NicheNet and CellChat analyses suggested that capVEC2 functions as an integrative hub for pro-angiogenic signaling, with prominent enrichment of the VEGF pathway, along with FGF, ANGPT, and Wnt signaling pathways. These signaling features are consistent with the angiogenic activation and expansion of capVEC2 during vascular remodeling. Notably, immunohistochemical and Western blot analyses have demonstrated increased VEGF expression in irradiated skin, with VEGF signals detected in scattered dermal fibroblasts [74], suggesting that microenvironment-derived VEGF may provide a paracrine angiogenic cue to capVEC2. Together, these findings indicate that capVEC2 represents a transient, radiation-induced endothelial state that integrates *Sp1*-driven transcriptional programs with VEGF-centered intercellular signaling to support vascular remodeling after irradiation.

Beyond vascular remodeling, recent studies have shown that endothelial cells in murine skin exhibit enrichment in immune-regulatory pathways following radiation, including IL-17 signaling, antigen processing and presentation, and cytokine–cytokine receptor interaction [75], indicating their involvement in inflammatory responses and immune modulation during tissue repair. A spatiotemporal single-cell analysis of human skin wound healing further highlighted the crucial role of interactions between endothelial cells, macrophages, and fibroblasts in coordinating repair processes [76]. Consistent with these findings, our study suggests that capVEC2, as a key endothelial population in post-irradiation skin, communicates with KER through a vascular–epidermal communication axis, potentially acting as a regulatory hub for multiple pro-reparative (e.g., TWEAK) and immune-modulatory signals (e.g., MHC-I). Through this coordinated signaling, capVEC2 helps to shape the microenvironment that facilitates epidermal repair and the restoration of tissue homeostasis integrating coordinated immune surveillance, regenerative processes, and inflammatory regulation. Moreover, prior studies have demonstrated that endothelial cells secrete cytokines such as IL-7 [77] and IL-6 [78], which may influence epidermal stem cell behavior, further supporting the link between capVEC2 and tissue regeneration. Together, these findings suggest that capVEC2 represents a transient, radiation-induced endothelial state that integrates immune modulation and pro-repair signaling to support epidermal repair and restore tissue homeostasis after irradiation.

Although this study systematically characterized the dynamic changes and regulatory features of skin vascular endothelial cells following radiation injury at single-cell resolution, several limitations should be acknowledged. First, this work primarily relied on single-cell transcriptomic analyses from a single irradiation model (10 Gy, male C57BL/6 mouse dorsal skin). While multiple complementary computational approaches provided cross-validation, direct experimental validation remains limited. Second, whether the temporal features of endothelial functional remodeling identified here are generalizable to other tissue types, radiation doses, sexes, or anatomical sites remains to be determined.

Collectively, we propose that capVEC2 represents a radiation-induced endothelial state that integrates Sp1-driven transcriptional programs with VEGF-centered intercellular signaling to support vascular remodeling.

## 4. Materials and Methods

### 4.1. Mice and Groups

Male C57BL/6 J mice (weight of 20 ± 2 g) were purchased from Beijing Vital River Laboratory Animal Technology Co., Ltd. (Beijing, China). All the mice were bred in a specific pathogen-free environment under conditions of constant temperature of 22 ± 1 °C, relative humidity of 60%, and regular dark-light schedule (lights on from 7 a.m. to 7 p.m.) at the Experimental Animal Center of the Beijing Institute of Radiation Medicine (Beijing, China). A total of 60 mice were used in this study, including 54 random mice receiving dorsal irradiation and 6 random mice as sham-irradiated controls. For irradiation, mice were anesthetized by intraperitoneal injection of 0.5% pentobarbital sodium (43 mg/kg body weight) and exposed to a single dose of 10 gray (Gy) dorsal irradiation using a ^60^Co γ-ray irradiator at a dose rate of 49.60 R/min and a source-to-skin distance of 2.5 m. The irradiation field was confined to the dorsal region (interscapular to lumbar dorsum), with lead bricks used to shield the rest of the body. Sham-irradiated control mice underwent identical anesthesia and positioning but were completely shielded [79]. For sham-irradiated controls, dorsal skin samples were collected immediately after the irradiation procedure. Irradiated mice were randomly assigned to nine groups, and dorsal skin samples were collected at 8 h, 16 h, and 1, 2, 3, 5, 7, 14, and 28 days post irradiation. For each mouse, full-thickness dorsal skin (~2 cm along the cranial-caudal axis and ~1 cm perpendicular to it) was collected after depilatory cream (Veet) application. For each time point, dorsal skin samples from six mice were pooled prior to library preparation and subjected to scRNA-seq.

### 4.2. Single-Cell RNA Sequencing (scRNA-Seq) Library Construction and Sequencing

The tissues were washed twice with 1× PBS and minced into approximately 1 mm^3^ fragments with sharp scissors and digested with enzyme mixtures including collagenase and DNase I at 37 °C. Then, the single cell suspensions were collected through a 40 μm strainer (MACS^®^ SmartStrainer 40 μm, Miltenyi Biotec, Bergisch Gladbach, Germany), and centrifuged at 400 *g* for 10 min, the supernatant was completely removed and cells were resuspended with 1 mL 1× PBS containing 0.05% BSA. Single-cell suspensions were processed through the 10x Genomics Chromium Controller (10x Genomics, Pleasanton, CA, USA). The libraries were constructed following the protocol outlined in the Chromium Single Cell 3′ Reagent Kits v3.1 (1000268, 10x Genomics, Pleasanton, CA, USA) User Guide. The libraries were pooled for sequencing using Illumina Novaseq X-25B sequencer with paired-end 150 bp reading strategy at CapitalBio Technology (Beijing, China).

### 4.3. scRNA-Seq Data Processing

The raw scRNA-seq data were mapped with CellRanger (version 8.0.0) [80] to a manually built mouse reference genome GRCm38 with the annotation file GENCODE (version 38). The output filtered gene expression matrices of each sample were analyzed by R software (v.4.3.1) with the Seurat (v.4.4.0) package [81]. To filter out low-quality cells, cells with fewer than 600 or more than 5000 detected unique molecular identifiers (UMIs) were removed. To filter out the dead or dying cells, the cells that had over 5% UMIs derived from mitochondrial genome were further removed. Potential doublets were detected using DoubletFinder (v.2.0.4) [82]. In total, 85,358 cells with 25,717 genes from 10 samples were retained for downstream analyses.

Data normalization was performed using ‘NormalizeData’ function in Seurat. For dimensionality reduction, the 2000 most variable genes were identified based on standardized variance using the ‘FindVariableFeatures’ function with the ‘vst’ method in Seurat. Principal component analysis (PCA) was then performed on the scaled expression matrix of these highly variable genes. To correct for batch effects across different samples, sample batch correction was conducted using the ‘RunHarmony’ function from the Harmony package (v.1.2.0) [83]. The Harmony-corrected embeddings were subsequently used for downstream analyses.

Cell clustering was performed using the ‘FindNeighbors’ and ‘FindClusters’ functions in Seurat. The t-distributed Stochastic Neighbor Embedding (t-SNE) was generated using the ‘RunTSNE’ function in Seurat for visualization. Unsupervised clustering of single cells was performed using the Louvain algorithm implemented in Seurat with a resolution of 1.5, yielding 40 clusters. Cluster-specific marker genes were detected using the ‘FindAllMarkers’ function. Genes were retained as markers when they satisfied the following thresholds: adjusted *p* value < 0.05, log2 fold change > 0.25, and expression in at least 25% of cells. Cell types were assigned by integrating the top marker genes of each cluster with established marker signatures reported for known skin cell populations. For detailed characterization of endothelial subsets, vascular endothelial cells and lymphatic endothelial cells were extracted and analyzed independently following the same analytical procedure, including data normalization, identification of highly variable genes, batch correction, dimensionality reduction, and graph-based clustering. The clustering resolution was set to 0.1 for vascular endothelial cells and 0.2 for lymphatic endothelial cells.

### 4.4. Differential Abundance Testing with Milo

Differential abundance analysis was conducted using the MiloR package (v.1.10.0) [24]. A k-nearest neighbor (KNN) graph was generated from the Harmony-corrected low-dimensional embedding of the processed Seurat object (k = 20, d = 20). The cell neighborhoods were defined using the ‘makeNhoods’ function with a sampling proportion of 0.1. The number of cells from each sample within each neighborhood was quantified using the ‘countCells’ function. Neighborhood distances were then calculated with the ‘calcNhoodDistance’ function based on the Harmony embedding (d = 20). Differential abundance testing between irradiation stages was performed using the ‘testNhoods’ function with a generalized linear model. *p* values were adjusted using the spatial false discovery rate implemented in MiloR, and neighborhoods with SpatialFDR < 0.1 were considered significantly differentially abundant.

### 4.5. Trajectory Analysis

Monocle 3 (v.1.3.1) [25] was applied to construct pseudotime trajectory of vascular endothelial cells. The venVEC was selected as a starting point of the pseudotime trajectory. The differentially expressed driving genes along the trajectory were determined using Moran’s *I* test in the ‘graph_test’ function, with the filtering criteria: q_value < 0.05 and morans_I > 0.15. Slingshot (v.2.10.0) [27] was applied to infer pseudotime by fitting simultaneous principal curves along the minimum spanning tree. RNA velocity analysis of vascular endothelial cells was carried out using the scVelo package (v.0.3.2) [26], which predicted the cell differentiation trajectory and its directionality based on the spliced and unspliced mRNA content.

### 4.6. Functional Enrichment Analyses

Functional enrichment analyses of the DEGs were performed using the R package clusterProfiler (v.4.10.1) [84] based on the gene sets of Gene Ontology (GO) or Kyoto Encyclopedia of Genes and Genomes (KEGG). The enriched terms with adjusted *p* < 0.05 were considered to be statistically significant.

### 4.7. Tip Signature Score

Tip signature was derived from the study published by Pan et al. [31]. The vascular endothelial cells were used to calculate the tip-like scores using the ‘AddModuleScore’ function in the Seurat package.

### 4.8. SCENIC and CellOracle Analyses

SCENIC analysis of vascular endothelial cells was performed using the pySCENIC (v.0.12.1) [32] The corresponding motif annotation file (mgi.v9.m0.001) and the cisTarget transcription factor ranking database (cisTarget.mm10.mc9nr.feather) were obtained from the pySCENIC repository (https://github.com/aertslab/pySCENIC (accessed on 19 March 2026)). Raw count expression matrices of vascular endothelial cells were used as input to infer gene regulatory relationships between transcription factors and candidate target genes. Gene modules were first inferred based on co-expression patterns and subsequently refined by motif enrichment analysis to identify transcription factor–target gene regulatory units (regulons). The activity of each regulon across individual cells was quantified using the AUCell algorithm.

To further explore transcription factor–driven regulatory dynamics, in silico perturbation analysis of gene regulatory networks was performed using CellOracle (v0.18.0) [46]. The regulatory influence of Sp1 was evaluated by simulating transcription factor perturbation, in which Sp1 expression was set to 0 to represent knockout conditions and increased to 1 to mimic overexpression beyond endogenous levels. The resulting simulated regulatory vector field was compared with the original developmental vector field, and a perturbation score (PS) was calculated to estimate the effect of Sp1 perturbation on cell-state transitions. Positive PS values indicated promotion of differentiation, whereas negative values indicated inhibition.

### 4.9. Cell-to-Cell Communication Analyses

NicheNet communication analysis was performed using the nichenetr package (v.2.2.0) [47]. The capVEC2 was defined as the receiver cell population, while all other cell types were considered as sender populations. NicheNet used mouse gene expression data from interacting cell populations as input and combined these data with a prior model that integrates existing knowledge of ligand-to-target signaling pathways. Ligand-receptor interactions and associated gene expression changes in capVEC2 were also predicted. CellChat (v.2.1.2) [57], which contains mouse ligand-receptor interaction databases, was used to construct the intercellular communication networks among different cell types. The CellChatDB.mouse database was applied to infer major signaling inputs and outputs across all cell types. Ligands and receptors expressed in more than 10 cells within a given cell cluster were retained for subsequent analyses.

### 4.10. Statistical Analyses

All data were analyzed and visualized by R (v.4.3.1) in this study. Wilcoxon test was used to assess the difference between groups in this study. Specifically, it was applied to compare tip scores among endothelial subpopulations in Figure 3D. *p* < 0.05 were considered statistically significant in all statistical tests. Visualization was done using the ggplot2 (v.3.5.1) R package.

## Data Availability

All data supporting the findings in this study are available upon request from the corresponding author. The data that support the findings of this study have been deposited into CNSA with accession number CNP0009041.

## Supplementary Materials

The following supporting information can be downloaded at: https://www.mdpi.com/article/10.3390/ijms27062879/s1.

## Abbreviations

The following abbreviations are used in this manuscript: IR, ionizing radiation; ROS, reactive oxygen species; scRNA-seq, single-cell RNA sequencing; Gy, gray; p.i., post-irradiation; LEC, lymphatic endothelial cell; capLEC, capillary lymphatic endothelial cell; collLEC, collecting lymphatic endothelial cell; vLEC, valve lymphatic endothelial cell; VEC, vascular endothelial cell; artVEC, arterial vascular endothelial cell; venVEC, venous vascular endothelial cell; capVEC, capillary vascular endothelial cell; FIB, fibroblast; KER, keratinocyte; HF, hair follicle cell; IC, immune cell; SMC, smooth muscle cell; Sch, Schwann cell; MEL, melanocyte; t-SNE, t-distributed Stochastic Neighbor Embedding; UMAP, Uniform Manifold Approximation and Projection; TF, transcription factor; RSS, regulon specificity score; GO, Gene Ontology; KEGG, Kyoto Encyclopedia of Genes and Genomes; UMI, unique molecular identifier; PCA, principal component analysis; GRN, gene regulatory network; PS, perturbation score.
