# Supplementary material for: Temporal Dynamics of Endothelium After Radiation Injury Reveal a Transient Pro-Angiogenic Capillary Subpopulation Associated with Skin Repair
Source: Int J Mol Sci. 2026 Mar 22;27(6):2879. doi: 10.3390/ijms27062879 (PMC13027251; doi:10.3390/ijms27062879)
Supplement: Supplementary file 1 [file ijms-27-02879-s001.zip › ijms-4182519-supplementary.pdf]

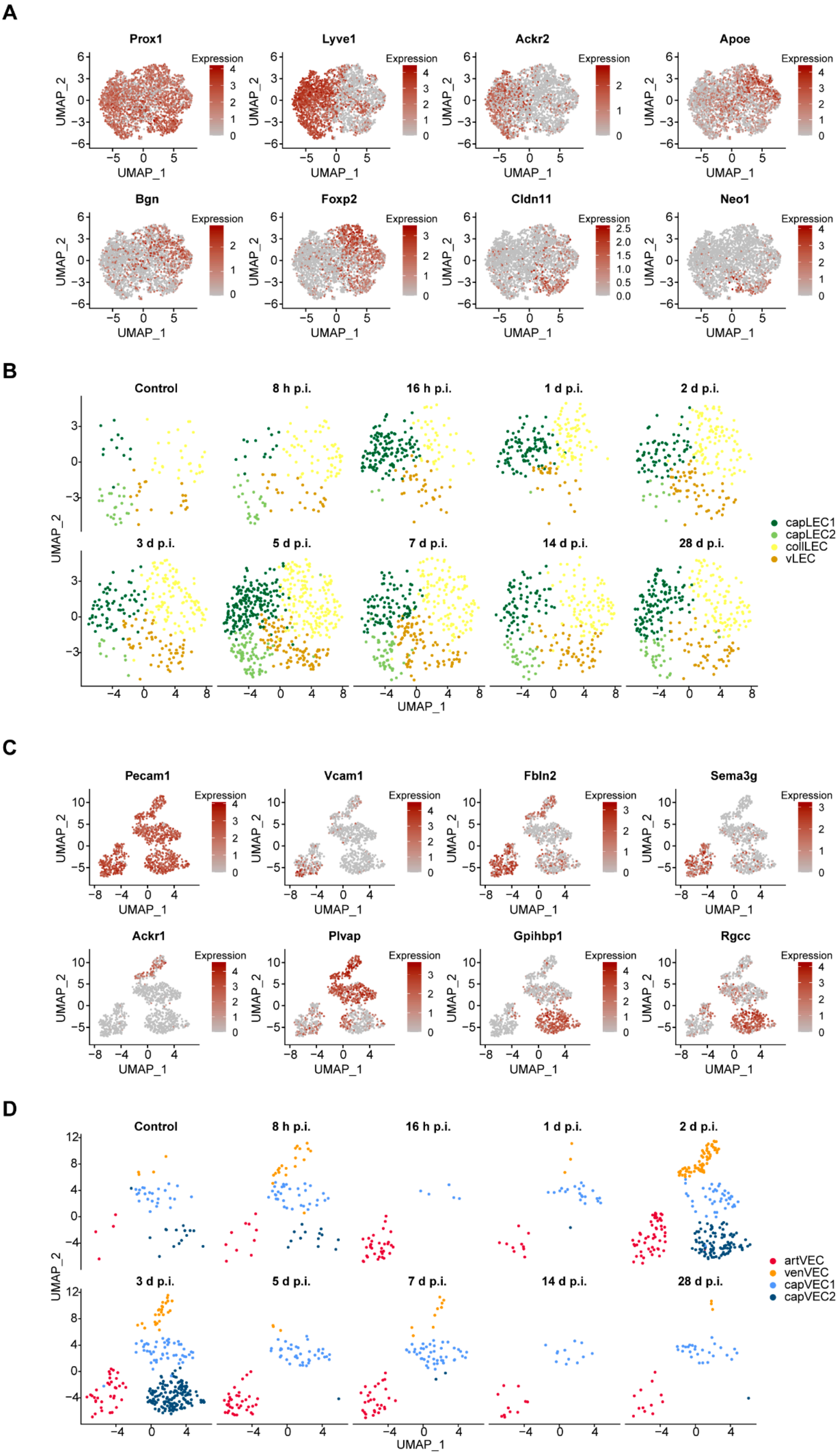
A
Prox1
Lyve1
Ackr2
Apoe
Bgn
Foxp2
Cldn11
Neo1
Expression
UMAP_1
UMAP_2
B
Control
8 h p.i.
16 h p.i.
1 d p.i.
2 d p.i.
3 d p.i.
5 d p.i.
7 d p.i.
14 d p.i.
28 d p.i.
capLEC1
capLEC2
collLEC
vLEC
UMAP_1
UMAP_2
C
Pecam1
Vcam1
Fbln2
Sema3g
Ackr1
Plvap
Gpihbp1
Rgcc
Expression
UMAP_1
UMAP_2
D
Control
8 h p.i.
16 h p.i.
1 d p.i.
2 d p.i.
3 d p.i.
5 d p.i.
7 d p.i.
14 d p.i.
28 d p.i.
artVEC
venVEC
capVEC1
capVEC2
UMAP_1
UMAP_2

Supplementary Figure S1. Additional visualization of endothelial cell heterogeneity and temporal dynamics post-irradiation.

(A) Feature plots showing the expression of representative marker genes for lymphatic endothelial cell (LEC) subpopulations, visualized on UMAP embeddings. **(B)** UMAP plots showing the distribution of LEC subpopulations across control and post-irradiation time points, with cells colored by LEC subtypes. **(C)** Feature plots showing the expression of representative marker genes for vascular endothelial cell (VEC) subpopulations, visualized on UMAP embeddings. **(D)** UMAP plots showing the distribution of VEC subpopulations across control and post-irradiation time points, with cells colored by VEC subtypes.

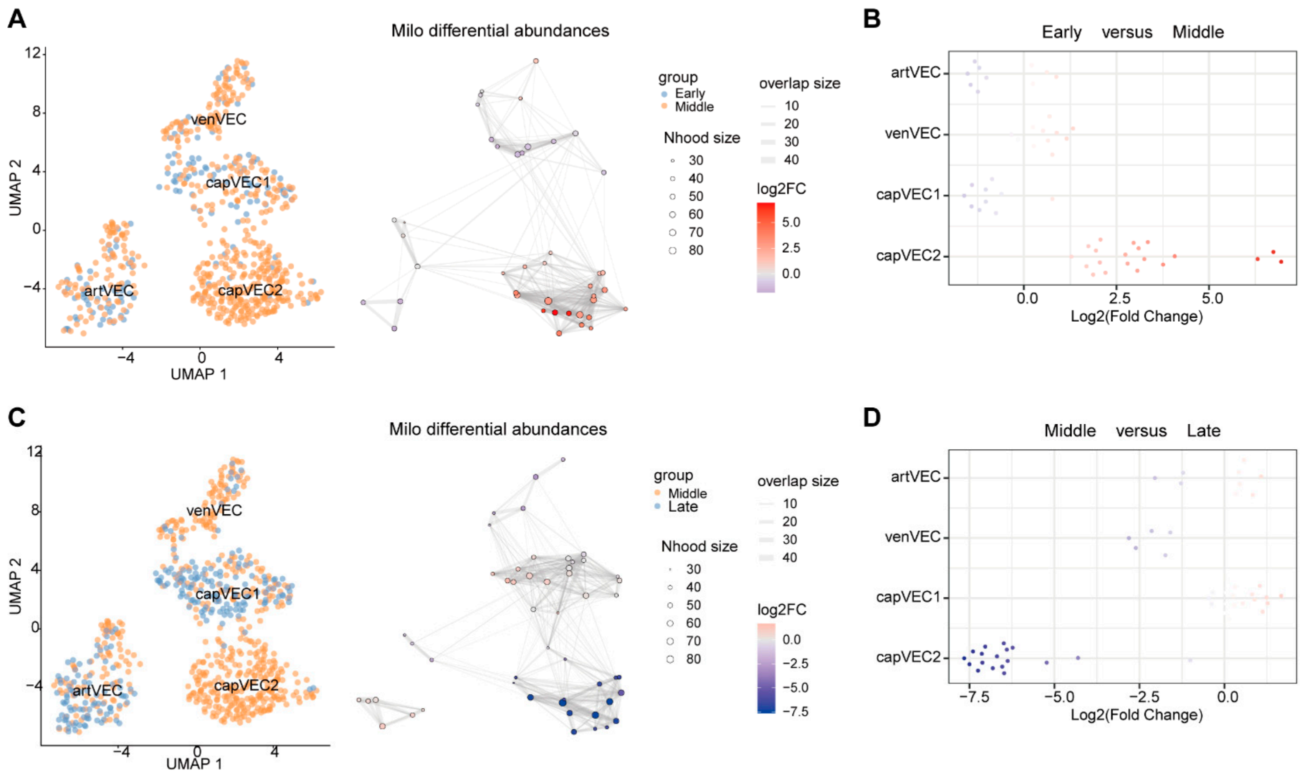

Supplementary Figure S2. Differential abundance analysis of vascular endothelial subpopulations across radiation response stages.

(A) Milo analysis comparing the early stage and middle stage. The left panel shows the UMAP embedding of vascular endothelial cells colored by stage. The right panel shows the graph representation of cell neighborhoods constructed by Milo. Each node represents a neighborhood of transcriptionally similar cells, with node size proportional to the number of cells in the neighborhood. Edges represent the overlap in cell membership between neighboring neighborhoods. Nodes are colored according to the $\log_2$ fold change ($\log_2$FC) in neighborhood abundance between stages. **(B)** Beeswarm plot showing the distribution of neighborhood differential abundance across endothelial subpopulations for the early stage versus middle stage comparison. Positive $\log_2$FC values indicate increased abundance in the middle stage relative to the early stage, whereas negative values indicate decreased abundance. **(C)** Milo analysis comparing the middle stage and late stage. **(D)** Beeswarm plot showing the distribution of neighborhood differential abundance across endothelial subpopulations for the middle stage versus late stage comparison. Positive $\log_2$FC values indicate increased abundance in the late stage relative to the middle stage, whereas negative values indicate decreased abundance.

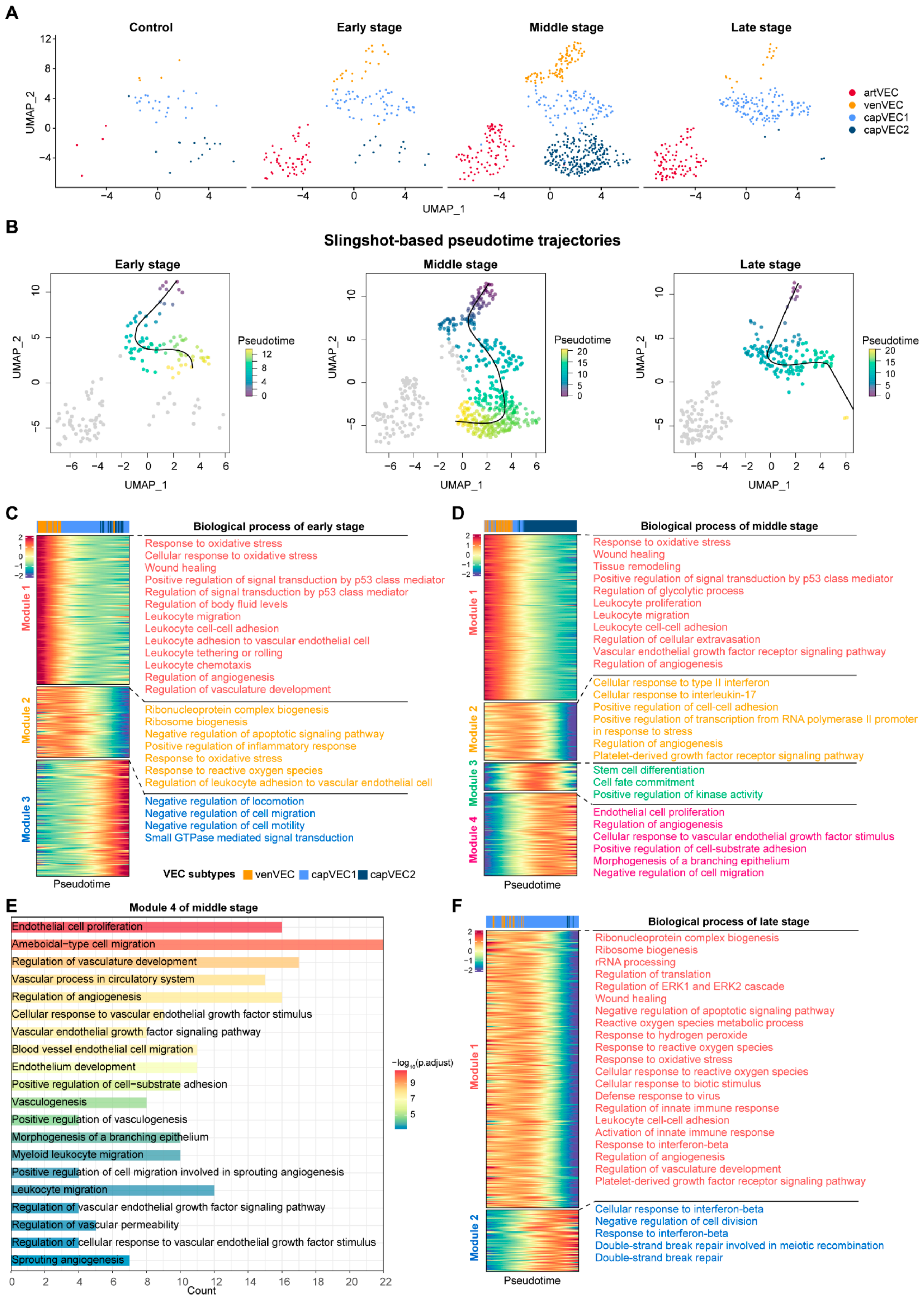
A
Control
Early stage
Middle stage
Late stage
UMAP_2
UMAP_1
artVEC
venVEC
capVEC1
capVEC2
B
Slingshot-based pseudotime trajectories
Early stage
Middle stage
Late stage
Pseudotime
UMAP_2
UMAP_1
C
Biological process of early stage
Module 1
Response to oxidative stress
Cellular response to oxidative stress
Wound healing
Positive regulation of signal transduction by p53 class mediator
Regulation of signal transduction by p53 class mediator
Regulation of body fluid levels
Leukocyte migration
Leukocyte cell-cell adhesion
Leukocyte adhesion to vascular endothelial cell
Leukocyte tethering or rolling
Leukocyte chemotaxis
Regulation of angiogenesis
Regulation of vasculature development
Module 2
Ribonucleoprotein complex biogenesis
Ribosome biogenesis
Negative regulation of apoptotic signaling pathway
Positive regulation of inflammatory response
Response to oxidative stress
Response to reactive oxygen species
Regulation of leukocyte adhesion to vascular endothelial cell
Module 3
Negative regulation of locomotion
Negative regulation of cell migration
Negative regulation of cell motility
Small GTPase mediated signal transduction
Pseudotime
VEC subtypes venVEC capVEC1 capVEC2
D
Biological process of middle stage
Module 1
Response to oxidative stress
Wound healing
Tissue remodeling
Positive regulation of signal transduction by p53 class mediator
Regulation of glycolytic process
Leukocyte proliferation
Leukocyte migration
Leukocyte cell-cell adhesion
Regulation of cellular extravasation
Vascular endothelial growth factor receptor signaling pathway
Regulation of angiogenesis
Module 2
Cellular response to type II interferon
Cellular response to interleukin-17
Positive regulation of cell-cell adhesion
Positive regulation of transcription from RNA polymerase II promoter in response to stress
Regulation of angiogenesis
Platelet-derived growth factor receptor signaling pathway
Module 3
Stem cell differentiation
Cell fate commitment
Positive regulation of kinase activity
Module 4
Endothelial cell proliferation
Regulation of angiogenesis
Cellular response to vascular endothelial growth factor stimulus
Positive regulation of cell-substrate adhesion
Morphogenesis of a branching epithelium
Negative regulation of cell migration
Pseudotime
E
Module 4 of middle stage
Endothelial cell proliferation
Ameboidal−type cell migration
Regulation of vasculature development
Vascular process in circulatory system
Regulation of angiogenesis
Cellular response to vascular endothelial growth factor stimulus
Vascular endothelial growth factor signaling pathway
Blood vessel endothelial cell migration
Endothelium development
Positive regulation of cell−substrate adhesion
Vasculogenesis
Positive regulation of vasculogenesis
Morphogenesis of a branching epithelium
Myeloid leukocyte migration
Positive regulation of cell migration involved in sprouting angiogenesis
Leukocyte migration
Regulation of vascular endothelial growth factor signaling pathway
Regulation of vascular permeability
Regulation of cellular response to vascular endothelial growth factor stimulus
Sprouting angiogenesis
Count
−log10(p.adjust)
F
Biological process of late stage
Module 1
Ribonucleoprotein complex biogenesis
Ribosome biogenesis
rRNA processing
Regulation of translation
Regulation of ERK1 and ERK2 cascade
Wound healing
Negative regulation of apoptotic signaling pathway
Reactive oxygen species metabolic process
Response to hydrogen peroxide
Response to reactive oxygen species
Response to oxidative stress
Cellular response to reactive oxygen species
Cellular response to biotic stimulus
Defense response to virus
Regulation of innate immune response
Leukocyte cell-cell adhesion
Activation of innate immune response
Response to interferon-beta
Regulation of angiogenesis
Regulation of vasculature development
Platelet-derived growth factor receptor signaling pathway
Module 2
Cellular response to interferon-beta
Negative regulation of cell division
Response to interferon-beta
Double-strand break repair involved in meiotic recombination
Double-strand break repair
Pseudotime

Supplementary Figure S3. Pseudotime dynamics and gene expression modules in vascular endothelial cells post irradiation.

(A) UMAP plots showing the distribution of VEC subpopulations across control, early, middle, and late stages post-irradiation. Cells are colored by VEC subtypes. early stage (8 h–1 d p.i.), middle stage (2 d–3 d p.i.), and late stage (3 d–28 d p.i.). **(B)** Slingshot-based pseudotime trajectories of VECs at the early, middle, and late stages, visualized on UMAP embeddings. Cells are colored according to pseudotime values. **(C)** Heatmap showing dynamic changes in gene expression along pseudotime at the early stage. Genes are grouped into modules based on their expression patterns using k-means clustering, and representative Gene Ontology biological processes (GOBP) associated with each module are shown on the right. **(D)** Heatmap showing dynamic changes in gene expression along pseudotime at the middle stage. Genes are grouped into modules based on their expression patterns using k-means clustering, and representative GOBP associated with each module are shown on the right. **(E)** Bar plot showing representative biological processes enriched in Module 4 of the middle stage. **(F)** Heatmap showing dynamic changes in gene expression along pseudotime at the late stage. Genes are grouped into modules based on their expression patterns using k-means clustering, and representative GOBP associated with each module are shown on the right.

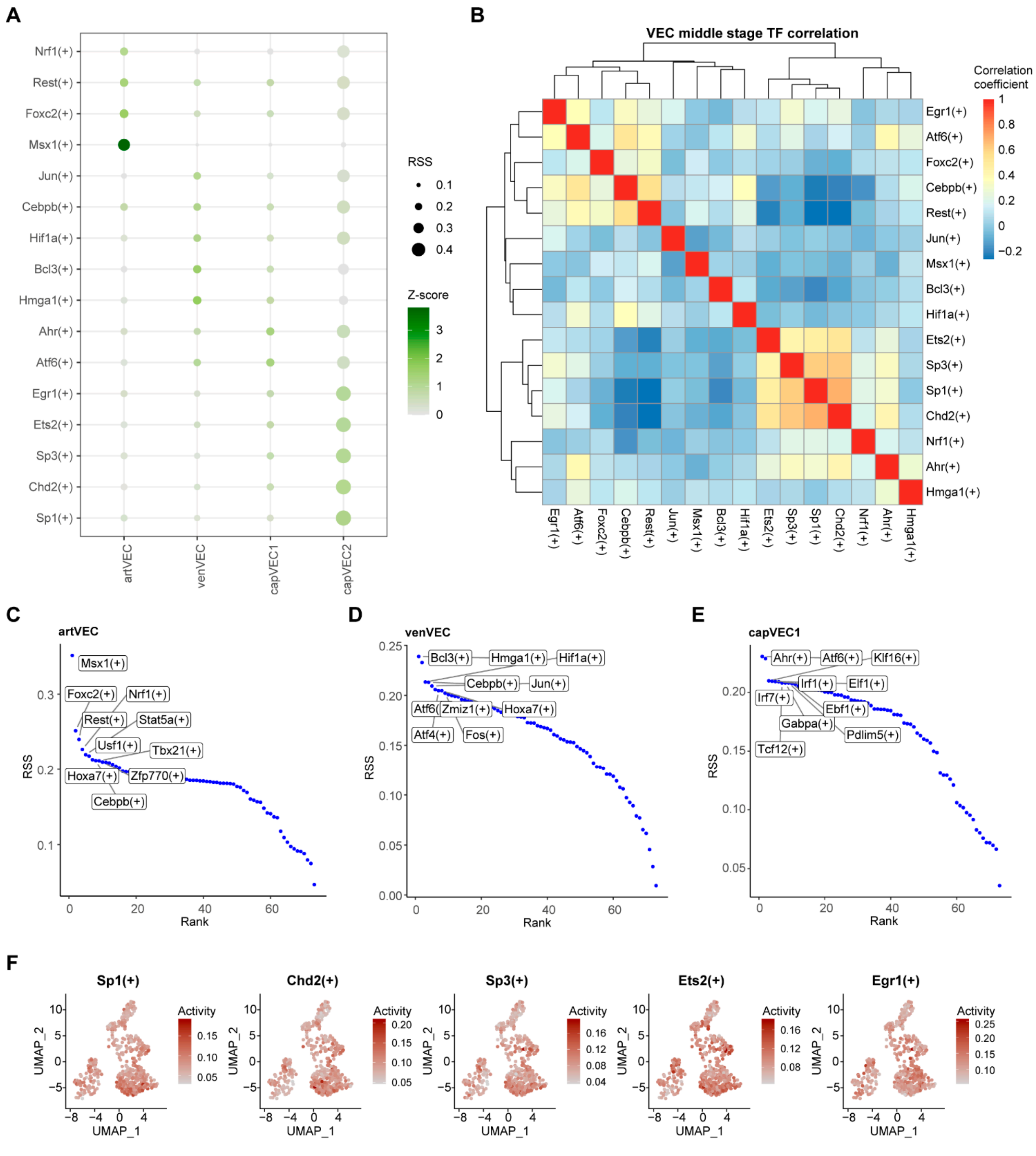

Supplementary Figure S4. Transcription factor analyses of vascular endothelial cell subpopulations during the middle stage.

(A) Dot plot showing regulon specificity scores (RSS) of top-ranked transcription factor (TF) regulons in VEC subpopulations. Dot size represents RSS, and color indicates scaled regulon activity (z score). **(B)** Heatmap showing Spearman correlation of TF regulon activities in VECs. **(C–E)** Scatter plots of the top 10 TF regulons ranked by RSS in artVEC (C), venVEC (D), and capVEC1

(E). **(F)** Feature plots showing the activity of representative TF regulons in capVEC2, visualized on UMAP embeddings.

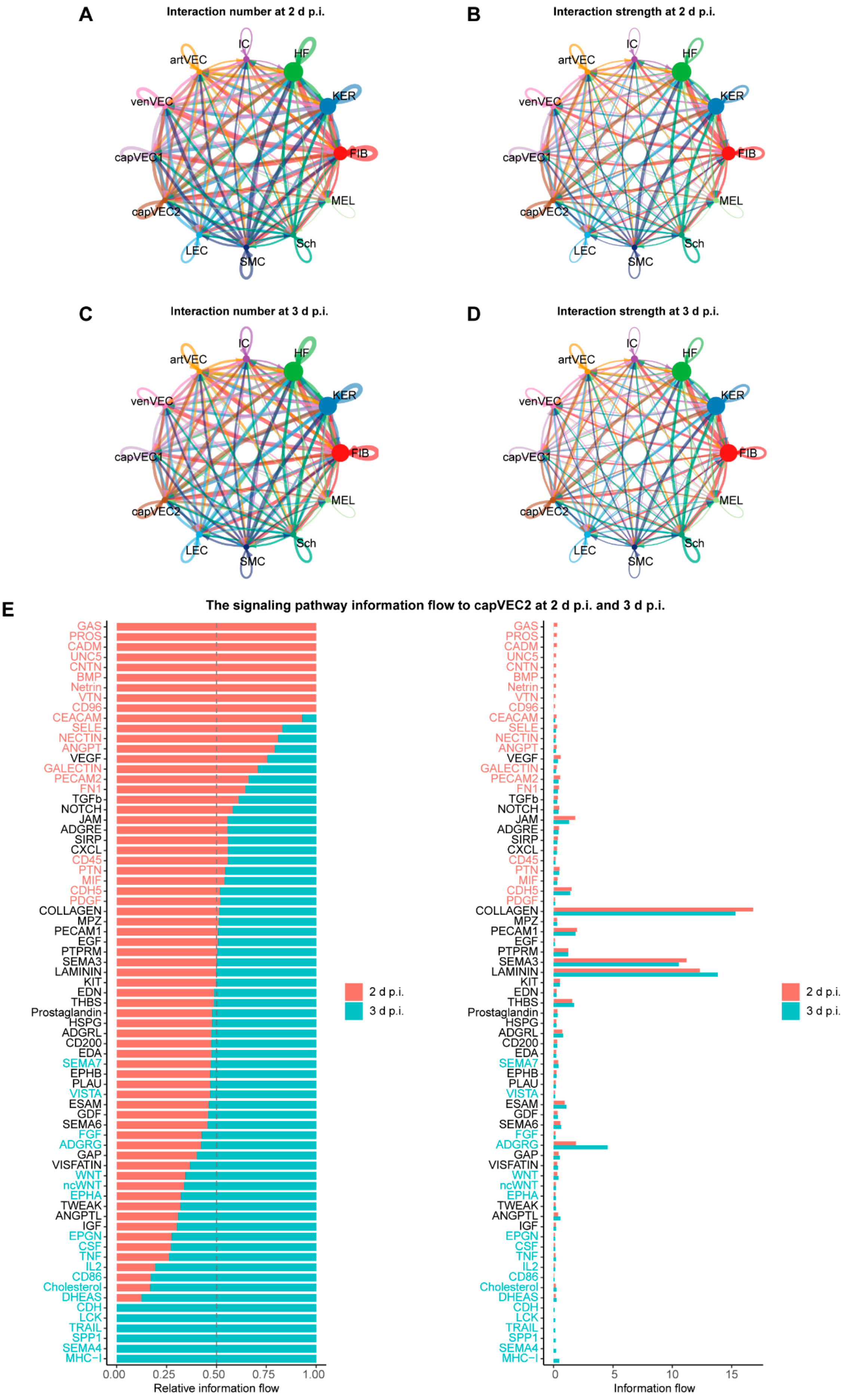
A
Interaction number at 2 d p.i.
B
Interaction strength at 2 d p.i.
C
Interaction number at 3 d p.i.
D
Interaction strength at 3 d p.i.
IC
HF
KER
FIB
MEL
Sch
SMC
LEC
capVEC2
capVEC1
venVEC
artVEC
E
The signaling pathway information flow to capVEC2 at 2 d p.i. and 3 d p.i.
GAS
PROS
CADM
UNC5
CNTN
BMP
Netrin
VTN
CD96
CEACAM
SELE
NECTIN
ANGPT
VEGF
GALECTIN
PECAM2
FN1
TGFb
NOTCH
JAM
ADGRE
SIRP
CXCL
CD45
PTN
MIF
CDH5
PDGF
COLLAGEN
MPZ
PECAM1
EGF
PTPRM
SEMA3
LAMININ
KIT
EDN
THBS
Prostaglandin
HSPG
ADGRL
CD200
EDA
SEMA7
EPHB
PLAU
VISTA
ESAM
GDF
SEMA6
FGF
ADGRG
GAP
VISFATIN
WNT
ncWNT
EPHA
TWEAK
ANGPTL
IGF
EPGN
CSF
TNF
IL2
CD86
Cholesterol
DHEAS
CDH
LCK
TRAIL
SPP1
SEMA4
MHC-I
0.00
0.25
0.50
0.75
1.00
Relative information flow
2 d p.i.
3 d p.i.
0
5
10
15
Information flow

Supplementary Figure S5. CellChat-based cell–cell communication landscape during the middle stage.

(A, B) Circular network plots showing the interaction number (A) and interaction strength (B) among major skin cell types at 2 d p.i.. Node size indicates cell number, and edge width reflects interaction number or strength. **(C, D)** Circular network plots showing the interaction number (C) and interaction strength (D) among major skin cell types at 3 d p.i.. **(E)** Bar plots showing signaling pathway information flow to capVEC2 at 2 d p.i. and 3 d p.i.. Left panel shows relative information flow, and right panel shows absolute information flow. Throughout this figure, FIB denotes fibroblast; KER, keratinocyte; HF, hair follicle cell; IC, immune cell; artVEC, arterial vascular endothelial cell; venVEC, venous vascular endothelial cell; capVEC1/2, capillary vascular endothelial subtypes 1 and 2; LEC, lymphatic endothelial cell; SMC, smooth muscle cell; Sch, Schwann cell; MEL, melanocyte.

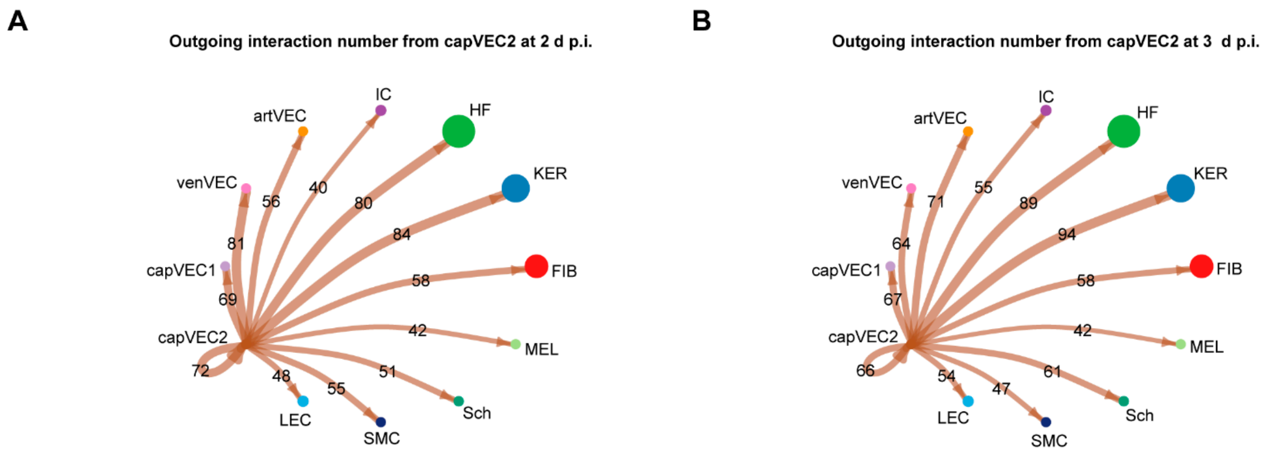

Supplementary Figure S6. CellChat-based outgoing interaction numbers from capVEC2 to different cell types during the middle stage.

(A, B) Outgoing interaction numbers from capVEC2 to different cell types at 2 d p.i. (A) and 3 d p.i. (B). Node size indicates cell number, and edge width reflects interaction number. Cell type abbreviations are consistent with those defined in supplementary figure 5.
